# Unexplored topics in intrahepatic cholestasis of pregnancy: A review and bibliometric analysis

**DOI:** 10.1097/MD.0000000000039972

**Published:** 2024-10-18

**Authors:** Qing Hu, Haiyan Yu

**Affiliations:** a Department of Obstetrics and Gynecology, West China Second University Hospital, Sichuan University, Chengdu, China; b Laboratory of Birth Defects and Related Diseases of Women and Children (Sichuan University), Ministry of Education, Chengdu, China.

**Keywords:** bibliometrics, CiteSpace, intrahepatic cholestasis of pregnancy, review, VOSviewer

## Abstract

To conduct a comprehensive bibliometric analysis of research published on intrahepatic cholestasis of pregnancy (ICP) and explore the related frontiers and critical issues concerning it, we searched the Web of Science Core Collection for ICP-related publications from the beginning of 2001 until August of 2023. CiteSpace and VOSviewer were utilized to evaluate the contribution and co-occurrence relationships of various countries and regions, institutes and so on to identify new frontiers and currently exciting topics. Our bibliometric analysis scrutinized 933 articles from 59 countries/regions. China has generated the largest number of publications (31.6% of the total), whereas Germany ranked first when it came to citations per publication. The Imperial College London ranked first with respect to publication output on ICP and betweenness centrality. The *Journal of Maternal-Fetal & Neonatal Medicine* was the journal with the highest plurality of papers. Authors such as Williamson, Beuers, Ulrich, and Shao were the most influential. Pregnancy, ursodeoxycholic acid, and ICP were principally noted in publications. Cluster analysis of the references that correlated with the 933 publications showed that they clustered into mortality, ABCB11, BSEP, MRP2, bile acid, and intrahepatic cholestasis. ICP is associated with adverse clinical outcomes for both the mother and fetus. This study provides a critical analysis of the current status and future research trends regarding ICP. It can serve as a useful reference, allowing researchers to conduct in-depth investigations into this promising field.

## 1. Introduction

Intrahepatic cholestasis of pregnancy (ICP) is a complication that occurs in 0.2% to 2% of pregnancies,^[1]^ and is characterized and diagnosed by pruritus and elevated maternal total serum bile acid concentrations in pregnant women.^[2,3]^ Stillbirth, as an adverse neonatal outcome, was demonstrated to be associated with maximal total bile acid concentration; and the prevalence of stillbirth in singletons varies from 0.28% to 3.44% when the serum bile acid varies between 40 and 100 µmol/L or more.^[4]^

Although ursodeoxycholic acid, which has a significantly lower bile acid hydrophobicity index,^[5]^ is widely used to treat ICP, a randomized controlled trial involving 605 women showed that treatment with ursodeoxycholic acid did not reduce adverse perinatal outcomes in women with ICP.^[3]^ Thus, the etiology and mechanism(s) underlying ICP remain poorly understood.^[6]^

Bibliometric analysis measures scientific progress across various disciplines of science and engineering,^[7]^ and it is a literature analysis method that evaluates the output and status of publications in a specific research field from both quantitative and qualitative perspectives.^[8]^ This type of analysis can be used to analyze, visualize, and identify key authors, institutions, countries, keywords, and other important information from published literature, thereby assisting researchers in quantitatively presenting the most cutting-edge research areas.^[9,10]^ Bibliometric analysis has thus far been used widely in medical research.^[11–14]^ Citations are created once an article references another peer-reviewed publication,^[15]^ and the number of citations of an article can quantitatively show its impact on the scientific community.^[16]^

Currently, no bibliometric analysis exists for research in ICP. We therefore conducted this study due to the need to elucidate the etiology and mechanism(s) underlying ICP, as well as to decrease the prevalence of adverse neonatal outcomes. We posit that our study will provide a critical analysis of the current status and future research trends with respect to ICP. We also expect that this bibliometric analysis will be a useful reference that will allow researchers to conduct deep-learning investigations into this promising field.

## 2. Materials and methods

### 2.1. Data sources and search strategy

For this analysis we systematically retrieved ICP reports from the Science Citation Index Expanded version in the Web of Science Core Collection. Publication date was restricted to include only papers from January 1, 2001, until August 14, 2023 in order to perform a comprehensive literature search of ICP. The systematic search strategy was designed as follows: (((TS = (pregnancy-related cholestasis)) or TS = (pregnancy-related cholestasis)) or TS = (obstetric cholestasis)) or (((TS = (pregnancy-related)) or TS = (intrahepatic of pregnancy)) and TS = (cholestasis)). Language was restricted to English and the document type was limited to original articles.

And then excluded articles that were not related to the research topic or duplicated. After article screening was completed, the subject and subtopic terms, title, abstract, author, institution, country, publication year, and other information of the included articles were exported in.

### 2.2. Data analysis

We applied Microsoft Office Excel 2019, VOSviewer (v 1.6.19), CiteSpace (v6.2.R2), and Bibliometrix (an R-tool for comprehensive science mapping analysis) (https://www.bibliometrix.org/home/) to analyze all 933 publications. VOSviewer (a bibliometric software) is a Java-based free software produced by Van Eck and Waltman of the Center for Science and Technology Studies (CWTS) at Leiden University in the Netherlands in 2009. VOSviewer is a software tool that is used for constructing and visualizing bibliometric networks.^[17]^ CiteSpace was developed by Professor Chen of Drexel University in the United States, and is also a Java application that supports visual exploration through knowledge discovery within bibliographic databases.^[18]^

**Figure 1.**
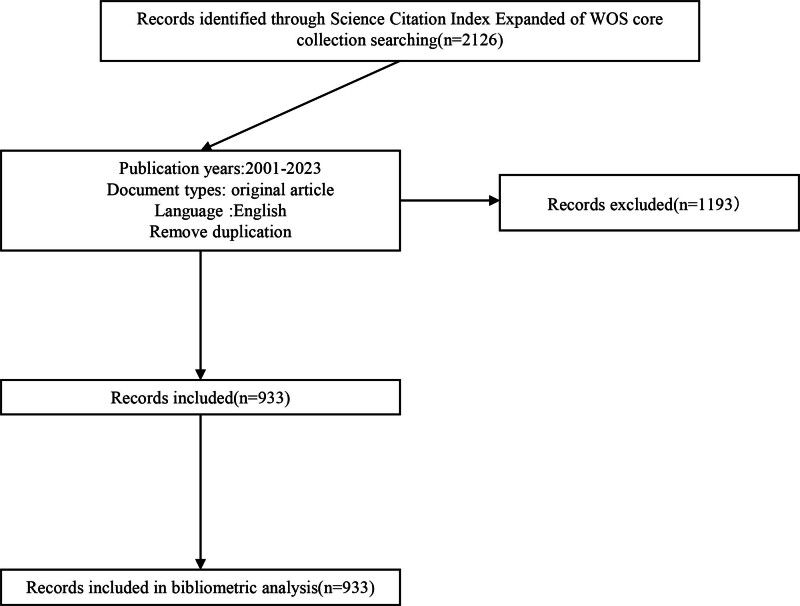
Flowchart of the search.

A total of 2126 documents were retrieved from the Science Citation Index Expanded of the Web of Science Core Collection. After we limited the publication year, document type, and language, 1193 documents were excluded. Finally, 933 documents met our inclusion criteria and were imported into the bibliometric analysis. The search details are presented as a flowchart in Figure 1. The retrieved data were collected on August 14, 2023.

## 3. Results

### 3.1. Annual publications

**Figure 2.**
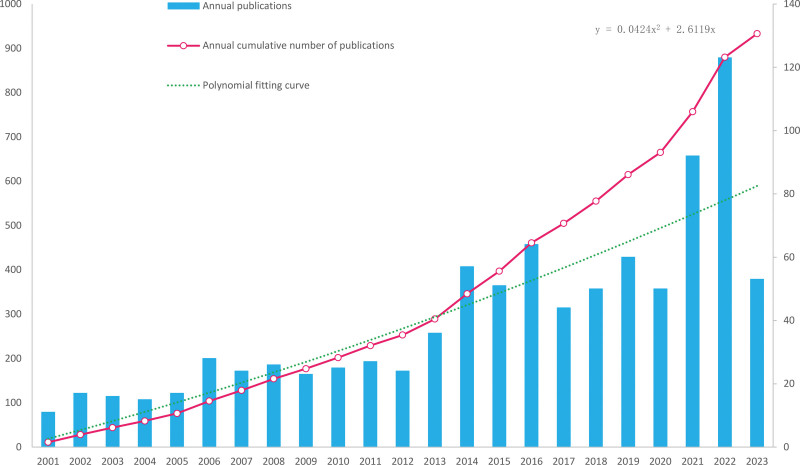
Annual publications and annual cumulative number of publications.

The bibliometric analysis of the number of publications over a period of time in a longitudinal analysis can reveal how particular groups within an intellectual structure emerge, grow, or fade away.^[19]^ Figure 2 shows the chronological distribution of publications by year as a bar graph. From 2001 to 2016, the annual number of publications increased slowly with gentle slopes, but remained relatively stable; from 2017 to 2022, the annual number of publications grew quickly, yet also exhibited shallow slopes. The cumulative number of publications by year is illustrated in Figure 2 as well, with an increased rate. The year 2019 was slow for publishing on this subject. However, 2014 and 2015 seem to have publication numbers higher than previous years and above the polynomial fitting curve.

### 3.2. Geographical distribution and cooperation between countries/regions

**Figure 3.**
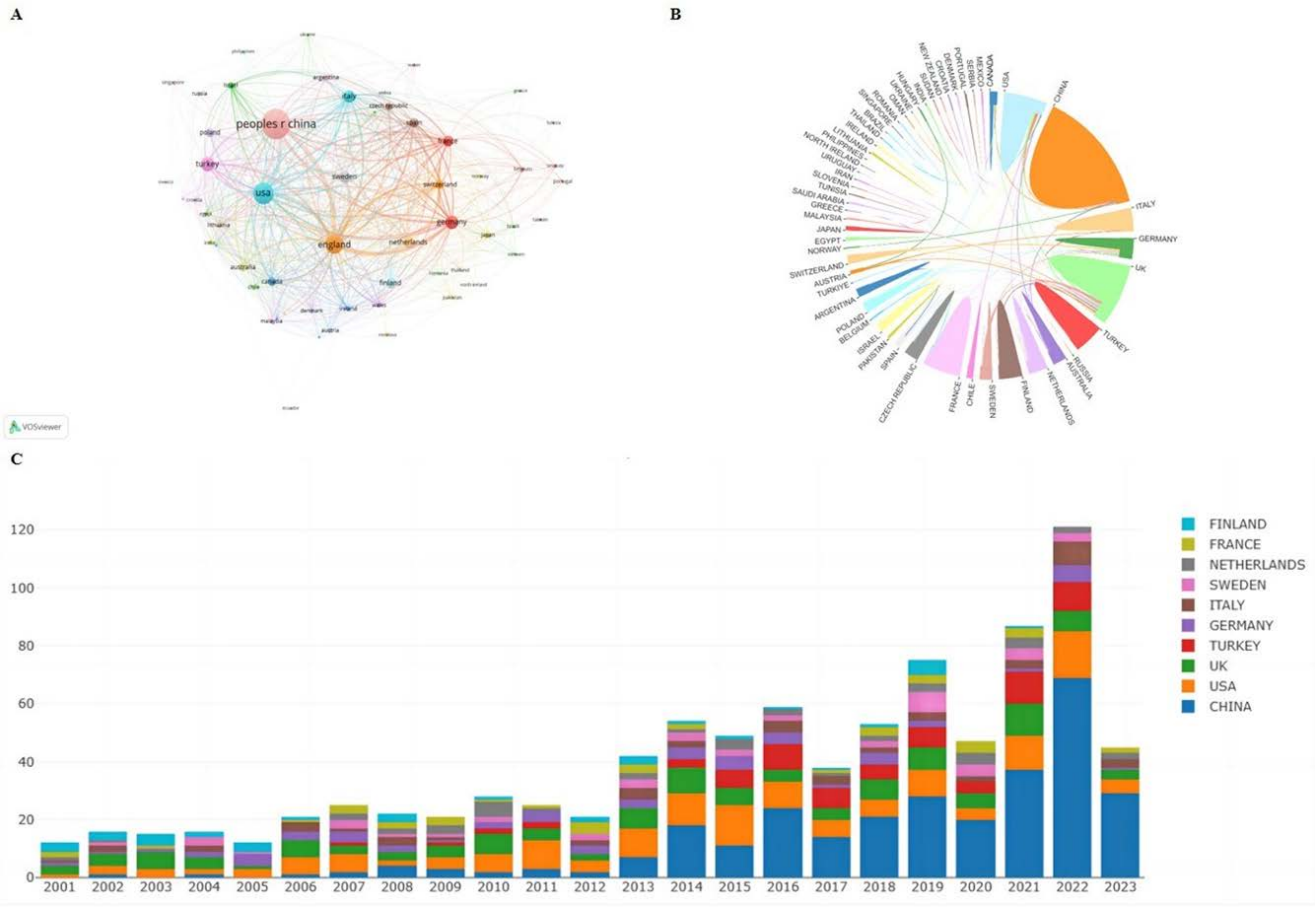
Contributions of various countries/regions to the research of intrahepatic cholestasis of pregnancy. (A) Countries/regions collaboration map of intrahepatic cholestasis of pregnancy. VOSviewer parameters were set as follows: the method (Linlog/modularity) and the minimal number of country documents were designated as 1; the circles denote the various countries, the size of the nodes represents the number of documents, and the thickness of the lines represents the number of connections between nodes. VOSviewer classified countries into different clusters and colored them according to the time course when they appeared. (B) The cooperation relationships of countries or regions. Circle denotes countries/regions, and the lines denote their collaborations. (C) The 10 high-output countries/regions and their output every year.

Regarding geographical distribution, 933 documents were published from 56 different countries and regions, with each country counted separately. We classified documents by country and visualized the spatial distribution by exploiting VOSviewer overlay visualization (Fig. 3A). Table 1 lists the top 10 countries with the most prolific publication rates. China had the largest total number of publications (295 of 933 publications for 31.6% of the total), far surpassing the UK (115/933 publications for 12.3%) and the USA rates (152/933 publications for 16.2%). Germany, however, ranked first when it came to citations. It was interesting that although China had the largest number of publications, the citations per publication lagged far behind other countries/regions. Figure 3B revealed that in the international collaboration network analysis, the USA and UK collaborated most frequently, followed by the USA and China. Figure 3C depicts the publications by year in the top 10 most productive countries/regions.

**Table 1:** Top 10 most productive countries/regions and their output.

Rank	Country/regions	Publications	Citations	Citations per publications
1	China	295	3090	10.47
2	USA	152	4292	28.23
3	UK	115	5544	48.21
4	Turkey	68	721	10.60
5	Germany	59	3917	66.38
6	Italy	49	1574	32.12
7	Sweden	44	2869	65.20
8	Netherlands	42	2325	55.36
9	France	39	1821	46.69
10	Finland	36	926	25.72

### 3.3. Analysis of prominent institutions and public sources

**Figure 4.**
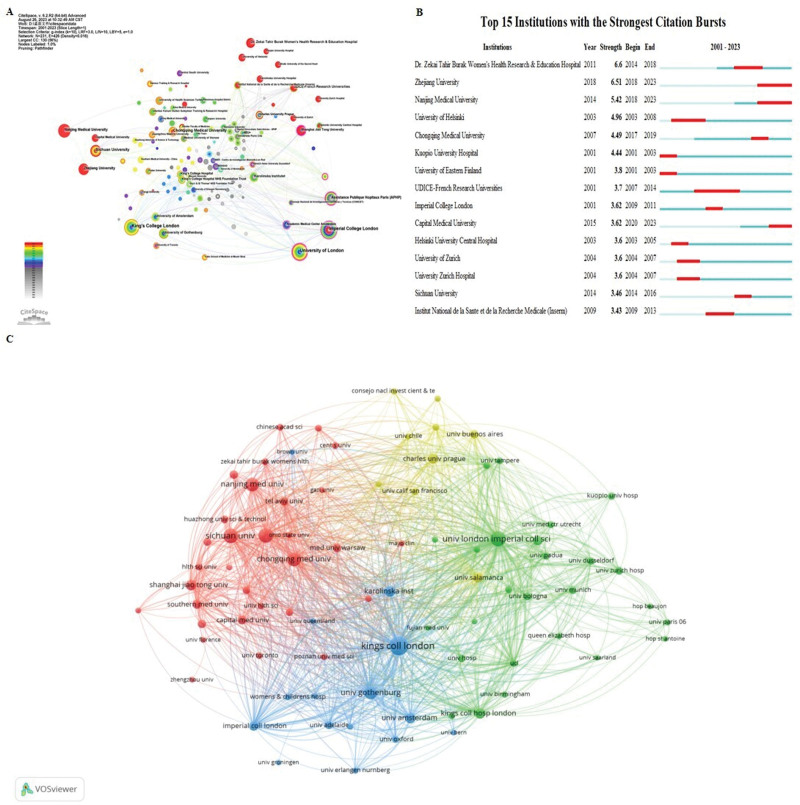
Contributions of different institutions to the research of intrahepatic cholestasis of pregnancy. (A) Visualization of institutions that conducted research on intrahepatic cholestasis of pregnancy. And the parameters were set as follows: time slice (2001–2023), year per slice (1), node type (organizations), and selection criteria (g index, *k* = 10). Nodes stand for institutions: the larger the node, the greater the output from the institution. Red nodes indicate that publications from the institution experienced a citation or frequency burst, and connecting lines of different colors represent different years. Some nodes shaped purple indicate high betweenness centrality, which is generally regarded as a milestone discovery. (B) The top 15 institutions with the strongest citation burst. The blue line denotes the timeline, and the red sections denote the burst interval, respectively, showing the beginning and end of the year, and the burst duration. (C) The bibliographic coupling analysis mapping of various institutions. We established the parameters as follows: method (association strength) and the minimal number of institution document were designated as 5. Circles denote different institutions and the lines represent the coupling relationship and its strength. All the nodes were classified by different colors according to time.

We identified that the 933 documents focusing on ICP were published by 403 different institutions (Fig. 4A). The institutional co-occurrence map organizations were constructed using CiteSpace. In terms of output by different institutions, the Imperial College London ranked first, followed by the University of London, Nanjing Medical University, and Assistance Publique-Hôpitaux de Paris. With regard to betweenness centrality, substantial contributions were made to the field of ICP by the Imperial College London (centrality = 0.06), UDICE-French Research Universities (centrality = 0.03), University of London (centrality = 0.02), Assistance Publiqe-Hopitaux de Paris (centrality = 0.02), and Sichuan University (centrality = 0.02). Figure 4B depicts the top 15 institutions with the strongest citation bursts and Figure 4C shows the bibliographic coupling analysis of different institutions using VOSviewer. The top institutions reflecting the most prolific publication rates are listed in Table 2, with Kings College London the most prolific with 52 documents published on ICP. However, the Imperial College of Science, Technology, and Medicine, University of London, ranked first based on citations per document. These data reveal that the work conducted by the Imperial College of Science, Technology, and Medicine, University of London, was rigorously effective. Core journals were identified by the analysis of bibliometric data from publication sources by employing the VOSviewer. We extracted the top 10 most productive journals, with details shown in Table 3.

**Table 2:** Top 15 most productive organizations and their output.

Rank	Organization	Documents	Citations per document	Total link strength
1	King's College London	52	42.13	1032
2	Imperial College of Science, Technology and Medicine, University of London	33	80.91	794
3	Zhejiang University	31	14.68	177
4	Sichuan University	31	12.16	145
5	Chongqing Medical University	28	13.79	141
6	Nanjing Medical University	28	8.29	89
7	University of Gothenburg	23	40.26	558
8	Karolinska Institute	17	71.00	380
9	Shanghai Jiao Tong University	17	9.88	110
10	King's College Hospital, London	16	31.38	234
11	University of Amsterdam	16	44.94	169
12	University of Salamanca	15	37.47	284
13	Charles University, Prague	15	22.07	116
14	University of Helsinki	14	23.57	62
15	Imperial College London	13	19.15	293

Total link strength in VOSviewer represents all links between a given node and other nodes, indicating how the entry interacted with other entries. The strength of a link is given by a nonnegative number. If one node has no links with other nodes, the total strength of the link equals zero.

**Table 3:** The top 10 most prolific journals and their influence.

Rank	Journal	JCR division	N (%)	Citations per publications	IF (2022)	JCI (2022)
1	*Journal of Maternal-Fetal & Neonatal Medicine*	Q2	43 (4.6%)	10	1.8	0.74
2	*Hepatology*	Q1	26 (2.7%)	116.6	13.5	3.01
3	*BMC Pregnancy and Childbirth*	Q2	26 (2.7%)	12.5	3.1	1.16
4	*Scientific Reports*	Q2	21 (2.2%)	19.8	4.6	1.06
5	*Archives of Gynecology and Obstetrics*	Q2	21 (2.2%)	12.7	2.6	0.83
6	*Placenta*	Q1	21 (2.2%)	17.4	3.8	1.09
7	*Journal of Obstetrics and Gynaecology Research*	Q3	20 (2.1%)	6.3	1.6	0.6
8	*PLoS One*	Q1	19 (2%)	32.2	3.7	0.91
9	*BJOG—An International Journal of Obstetrics and Gynaecology*	Q1	16 (1.7%)	46.6	5.8	2.01
10	*World Journal of Gastroenterology*	Q2	16 (1.7%)	22.1	4.3	0.82

Journal Citation Indicator is a measure of the average Category Normalized Citation Impact (CNCI) of citable items (articles and reviews) published by a journal over a recent 3-year period. It is used to facilitate the evaluation of journals based on other metrics in addition to the Journal Impact Factor.

**Figure 5.**
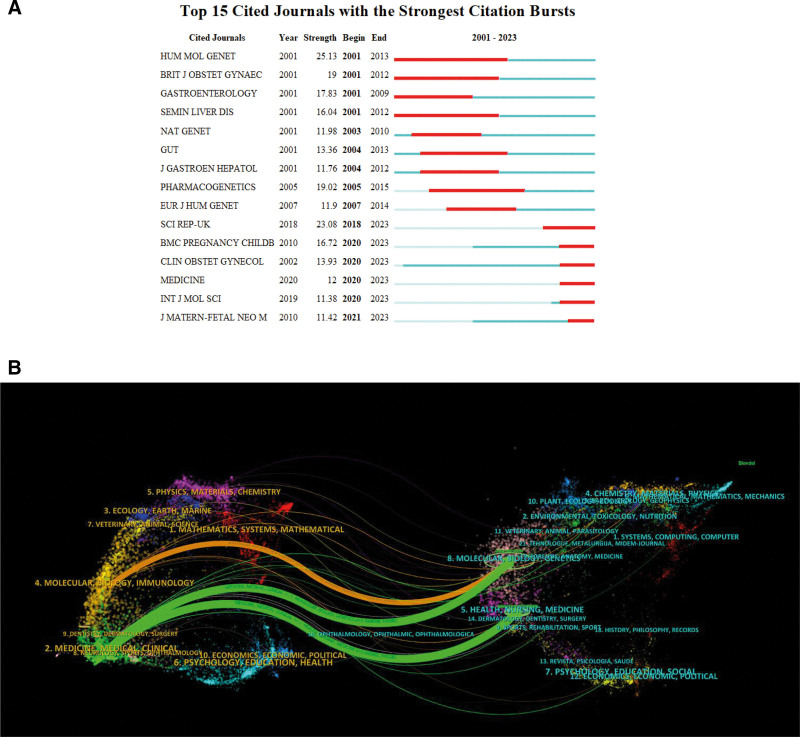
Cited journals exhibiting the strongest citation bursts and a dual-map overlay of the journals.

After analyzing bibliographies, we identified the top 15 most cited journals using CiteSpace, as illustrated in Figure 5A. Journals such as *Scientific Reports* (IF2022: 4.6), *BMC Pregnancy and Childbirth* (IF2022: 3.1), *Clinical Obstetrics and Gynecology* (IF2022: 1.5), *Medicine* (IF2022: 1.6), *International Journal of Molecular Sciences* (IF2022: 5.6), and *Journal of Maternal-Fetal and Neonatal Medicine* (IF2022: 1.8) have published increasingly significant articles in recent years. Furthermore, as illustrated in Figure 5B, we constructed a dual-map overlay of the journals to analyze the relationships between subject categories and ICP. We thereby uncovered the 3 most important paths. The 2 green citation paths indicated that research in Molecular Biology, Genetics, Health, Nursing, and Medicine were frequently cited by Medicine/Medical/Clinical journals; and the orange citation path indicated that research in Molecular Biology and Genetics journals were frequently cited by Molecular/Biology/Immunology journals.

### 3.4. Analysis of co-authorship and the most productive authors

**Figure 6.**
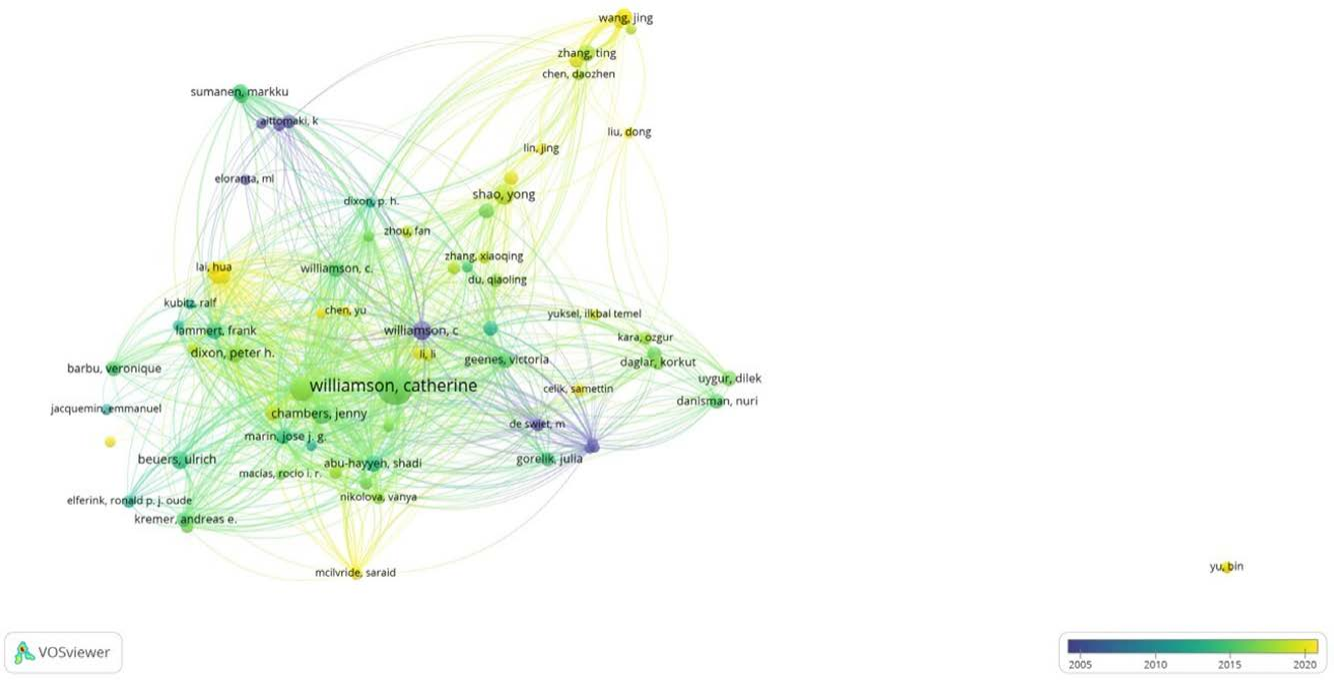
Authors with more than 5 publications on intrahepatic cholestasis of pregnancy. The circles denote authors and the lines represent their operations. Authors in blue published early than those in yellow.

Additionally, we analyzed author and coauthor information using VOSviewer. A total of 933 publications were produced by a total of 4801 authors, and our parameters were set as: method of no normalization and authors published ≥5 documents on ICP; the results were visualized using an overlay visualization (Fig. 6). The visualization identified 85 authors, with Catherine Williamson, Ulrich Beuers, and Shao Yong emerging as the most influential. We also noted that authors like Jing Wang, Jin Lin, and Sarid Mcilvride have recently published articles and may lead ICP research in the future.

The top 10 core authors are listed in Table 4. Among the top 10 most productive authors, 4 authors from the UK published 108 articles on ICP, accounting for 11.58% of the 933 documents. Of the top 10 most productive authors, Catherine Williamson published 75 articles and her H-index was 25, whereas Ulrich Beuers only conducted 13 studies on ICP but had an H-index of 71. This indicated that articles written by Ulrich Beuers proved to be the most scientifically effective in terms of publication citation.

**Table 4:** The top 10 most productive authors and their influence.

Rank	Author	Affiliations	Documents	Citations	H-index
1	Williamson, Catherine	King’s College London, Imperial College London	75	4123	25
2	Marschall, Hanns-Ulrich	Sahlgrenska University. Hospital University of Gothenburg	23	878	66
3	Shao, Yong	Chinese Academy of Agricultural Sciences Minister Agricultural & Rural Areas	17	282	24
4	Chambers, Jenny	Christ Hospital—Ohio Prairie Education & Research Cooperative	15	913	16
5	Dixon, Peter H	King’s College London, Imperial College London	13	756	8
6	Beuers, Ulrich	Amsterdam University Med Center, University of Amsterdam	13	930	71
7	Lammert, Frank	University Medical Center, Saarland Hannover Medical School	11	448	70
8	Zhang, Ting	Changzhou University, Key Lab High End Struct Mat, Jiangsu Province	11	140	13
9	Abu-Hayyeh, Shadi	King’s College London, Imperial College London	10	523	16
10	Geenes, Victoria	King’s College London, North West Thames Deanery	10	846	12

### 3.5. Analysis of keywords

**Figure 7.**
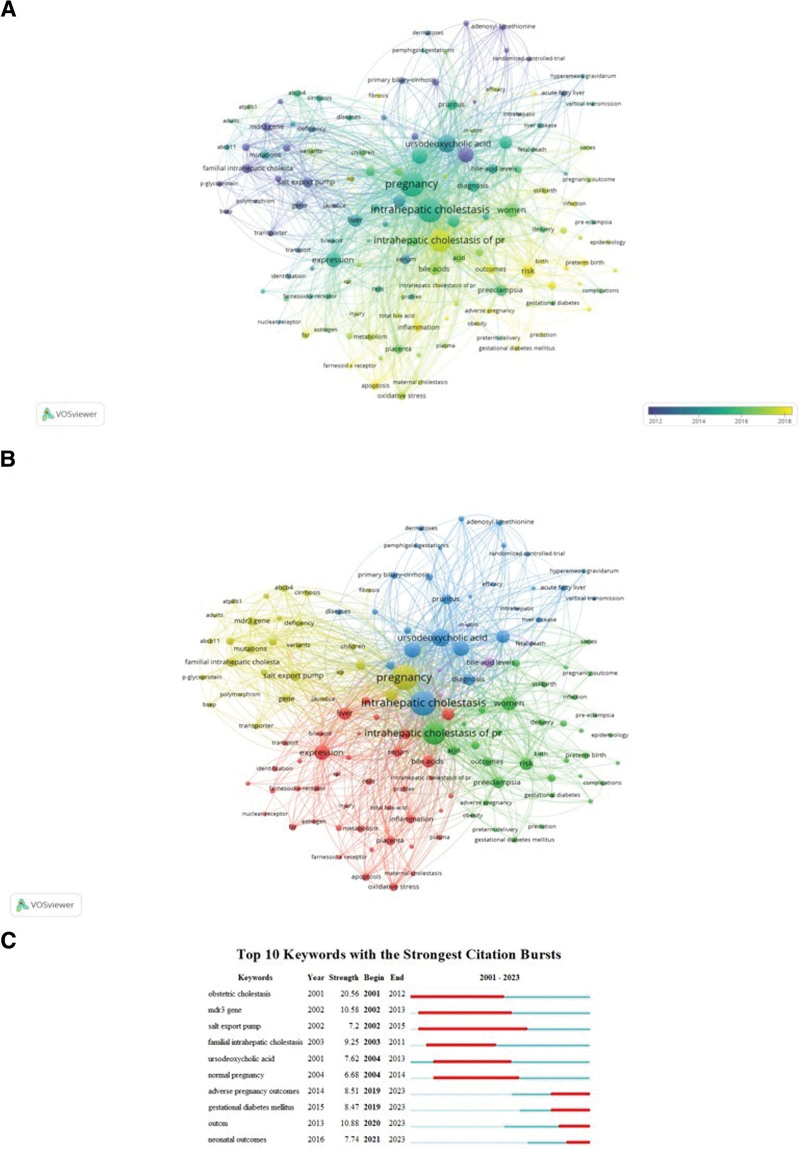
The keyword mapping of intrahepatic cholestasis of pregnancy. (A) The 136 keywords appearing more than 10 times. The circles denote different keywords and the lines denote their co-occurrence; colors were assigned in accordance with time. (B) Cluster analysis of keywords. The parameters were set as: method with association of strength and minimal number of occurrences of a keyword set at 10. The 136 keywords appearing over 13 times fell into 5 clusters based on colors: clusters 1, 2, 3, 4, and 5 are red, green, blue, yellow, and purple, respectively. The node size denotes the occurrence frequency and the lines denote their co-occurrence. (C) Top 10 keywords with the strongest citation burst. The blue line denotes the timeline, and the red sections denote the burst interval, respectively, showing the beginning and end of the year, and the burst duration.

In total, we extracted 3138 unique keywords from 933 documents and used network visualization in VOSviewer to display their co-occurrence. The parameters were set as: method with an association of strength and minimal number of occurrences of a keyword at 10. Results are shown in Figure 7A. One hundred thirty-six keywords occurred ≥10 times, with the greatest occurrences shown for intrahepatic cholestasis, pregnancy, ursodeoxycholic acid, and intrahepatic cholestasis of pregnancy. Keywords that included outcomes, risk, preterm birth, prediction, inflammation, farnesoid × receptor, stillbirth, and acute fatty liver appeared in recent years, indicating that research on ICP focused on adverse maternal-fetal outcomes: but also that its mechanisms remain poorly defined. Figure 7B is a network visualization of keywords using VOSviewer. The top 3 keywords in cluster 1 were expression, liver, and bile acids; the top 3 keywords in cluster 2 were ICP, women, and risk; intrahepatic cholestasis, ursodeoxycholic acid, and obstetric cholestasis were the top 3 keywords in cluster 3; pregnancy, disease, and salt export pump were the top 3 in cluster 4; and in cluster 5 the top 3 keywords were bile acid levels, fetal death, and model. Figure 7C describes the top 10 keywords with the most significant citation bursts. Adverse pregnancy outcomes, gestational diabetes mellitus, outcomes, and neonatal outcomes were the trendiest areas in the ICP research field.

### 3.6. Co-cited analysis of reference

**Figure 8.**
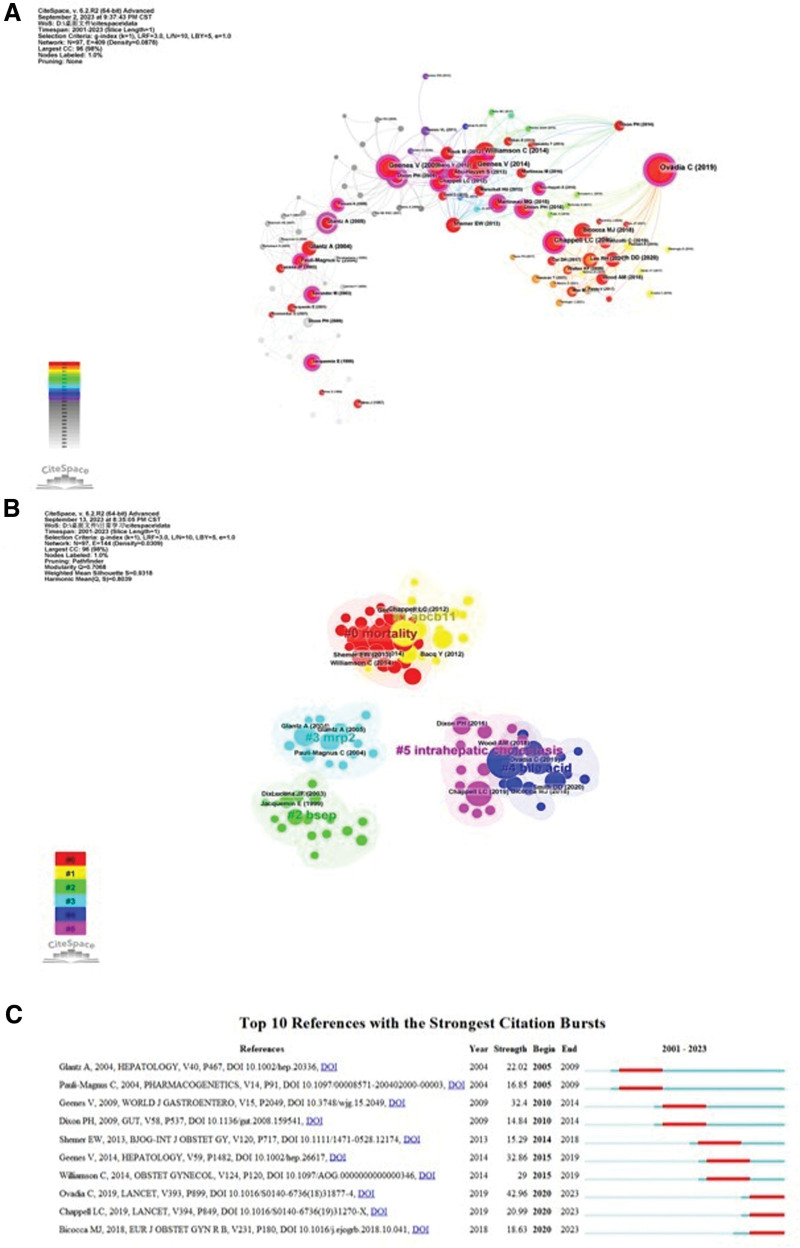
Visualization of co-cited references on intrahepatic cholestasis of pregnancy. (A) Network visualizations diagram of cited references. The parameters were set as time slice (2001–2023), year per slice (1), node type (reference), and selection criteria (g index, *k* = 1); nodes designate the references: the larger the node, the more frequently the reference was cited. Red nodes indicate references with a burst of being cited, and connecting lines represent their mutually cited relationship. Some nodes are shaped purple and represent their high betweenness centrality, which was generally regarded as a large breakthrough in ICP research. (B) The clustered network map of ICP-related co-cited references. (C) The 10 references having the strongest citation burst. The blue line denotes the timeline, and the red sections denote the burst interval, respectively, showing the beginning and end of the year, and the burst duration. ICP = intrahepatic cholestasis of pregnancy.

The 933 articles on ICP contained 17,772 references, and their co-citation relationships were analyzed using CiteSpace. The results are illustrated in Figure 8A. Figure 8B displays the relevant 6 clusters as divided by CiteSpace: mortality, abcb11, bsep, mrp2, bile acid, and intrahepatic cholestasis; Figure 8C depicts the top 10 references with the strongest citation bursts.

## 4. Discussion

The chronological trend we observed signifies a significant achievement, enabling us to draw a roadmap for this field of scientific endeavor. In 2014, 2 publications were cited 249 and 261 times, respectively. In the former, the authors reviewed the etiology, diagnosis, and management of ICP,^[20]^ while the latter was a prospective population-based case–control study that revealed significantly increased risks of adverse perinatal outcomes (including stillbirth) in ICP pregnancies.^[21]^ Beuers et al^[22]^ reviewed and delineated nuclear receptors (including farnesoid X receptor, retinoid X receptor, peroxisome proliferator-activated receptor-𝛼, and pregnane X receptor), membrane receptors (such as fibroblast growth factor receptor 4 and apical sodium bile acid transporter); and 23-C homologue of UDCA: all of which are now being investigated as promising targets for therapeutic interventions in cholestatic disorders. The review article by Beuers et al has been cited 329 times. In 2019, Ovadia et al^[4]^ performed a meta-analysis and concluded that most women with ICP who manifested bile acid concentrations below 100 µmol/L could be reassured that their risk of stillbirth was similar to that of pregnant women in the general population. These results are in accordance with the searching result in WOS(SCIE) database on the highly cited publications of ICP. Furthermore, a RCT published in 2019 was also highly cited with 143 times, which stated that treatment with ursodeoxycholic acid does not reduce adverse perinatal outcomes in ICP women.^[3]^ But in 2014, 2015, and 2019 we noted a slow rate of publication on ICP. The reason may be related to the much of the pathogenesis of this disorder is not fully understood^[23]^ and the current intervention has been analyzed with no favorable effect on pruritus and other symptoms in ICP patients.^[24]^ So, the potential mechanism or effective treatment still behind the veil and the studies need new breakthrough.

As for geographical distribution (Table 1), it is worth noting that China has taken the lead in publication output in ICP research. However, the citations per publication from China was still far below that of other countries such as Germany, Sweden, the Netherlands, and the UK. Regarding research institutions, 4 of the top 15 most-productive universities were in the UK (Table 2), with King’s College London ranking first among them. This is also in consistent with the results depicted in Table 4: 4 of 10 most productive authors are from the King’s College London. And 3 of 5 highly cited publications are affiliated with King’s College London.^[4,20,21]^ Thus, we concluded that the UK has contributed greatly to the academic impact and renown of ICP research. The USA and UK collaborated most frequently, followed by the USA and China. From this analysis, we noted that cooperativity may be strengthened among countries such as the UK, USA, Germany, Sweden, Netherlands, and China.

For the most productive journals, the top 10 journals with the most publications in ICP are depicted in Table 3. Hepatology received the largest number of co-citations, showing the significant influence exerted by this journal. The most recent impact factor for Hepatology (which was created in 1981) was 13.5, and it currently has an H-index of 361. The newest article on ICP published by Hepatology since 2020 was written by Li et al,^[25]^ in which these authors conducted a nationwide population-based study in Denmark, and concluded that co-twins and first-degree relatives of ICP patients were at an ~5- and ~2.5-fold increased risk of ICP, respectively. The most cited article on ICP published by Hepatology was a prospective population-based case-control study written by Geenes,^[21]^ which demonstrate significant increased risks of adverse perinatal outcomes, including stillbirth.

We performed an analysis of keywords to investigate research trends and found that the focus of ICP has changed drastically. In general, we observed that the critical research issues and public interest changed in 2 major aspects: from the mechanism(s) underlying ICP effects (such as the salt export pump, genetic basis, ABCB4/11, and p-glycoprotein) to the clinical aspects (such as stillbirth, preterm birth, and adverse pregnancy) (Fig. 7A). This change was consistent with keywords with strongest citation bursts (Fig. 7C). Articles published recently concentrated on the 2 aforementioned aspects as well.^[26–31]^ Although our analysis of keywords revealed that the mechanism(s) regulating ICP remains elusive, we were reminded that assessing ICP’s negative effects on perinatal outcome might constitute a novel avenue toward better understanding ICP.

We conducted cluster analysis and assessed citation bursts for the visualization and analysis of references. There were 6 clusters as analyzed by CiteSpace: mortality, ABCB11, BSEP, MRP2, bile acid, and intrahepatic cholestasis, and aspects of these clusters follow. We noted, for example, that the intrauterine and neonatal mortality rate was approximately 0.5%^[32]^ in ICP pregnancies. ABCB11 is a gene that encodes the bile salt export pump (BSEP) protein that is responsible for transporting bile salts from liver cells into the bile ducts for excretion.^[33]^ Multidrug resistance protein 2 (MRP2) is a multidrug resistance protein that is involved in the excretion of bilirubin, and its loss can lead to hyperbilirubinemia and acute liver failure.^[34]^ The relationship between ABCB11 (BSEP) and MRP2 is also reflected in the fact that they both contribute to the transport of bile salts from liver cells into the bile ducts. While BSEP specifically transports bile salts, MRP2 (also known as ABCC2) is involved in the transport of various organic anions, including bile salts.^[35]^ We hypothesize that these genes may comprise a novel target for the treatment of ICP in the future.

## 5. Conclusions

We expect that this bibliometric analysis will assist researchers in understanding the unexplored aspects and exciting and novel topics in intrahepatic cholestasis of pregnancy. The UK contributed greatly to the academic impact and renown of ICP research, and China is now taking the lead in publication output in this research field. The work conducted by the Imperial College of Science, Technology, and Medicine, University of London, was also significant. The journals *Scientific Reports* (IF2022:4.6), *BMC Pregnancy and Childbirth*, *Clinical Obstetrics and Gynecology*, *Medicine*, *International Journal of Molecular Science*, and *the Journal of Maternal-Fetal and Neonatal Medicine* showed prolific publication on ICP. Williamson, Beuers, and Shao were the most influential authors. Adverse pregnancy outcomes, gestational diabetes mellitus, outcomes, and neonatal outcomes were the trendiest and most popular topics. We deemed the indices of mortality, ABCB11, BSEP, MRP2, bile acid, and intrahepatic cholestasis to be at the frontiers of research on ICP.

## Author contributions

**Conceptualization:** Haiyan Yu.

**Data curation:** Qing Hu.

**Formal analysis:** Qing Hu.

**Funding acquisition:** Haiyan Yu.

**Investigation:** Qing Hu.

**Methodology:** Qing Hu.

**Project administration:** Haiyan Yu.

**Resources:** Qing Hu.

**Software:** Qing Hu.

**Supervision:** Haiyan Yu.

**Validation:** Haiyan Yu.

**Writing – original draft:** Qing Hu.

**Writing – review & editing:** Haiyan Yu.

## References

[1] Palmer KR Xiaohua L Mol BW. Management of intrahepatic cholestasis in pregnancy. Lancet. 2019;393:853–4. 30773279 10.1016/S0140-6736(18)32323-7

[2] Vasavan T Deepak S Jayawardane IA. Fetal cardiac dysfunction in intrahepatic cholestasis of pregnancy is associated with elevated serum bile acid concentrations. J Hepatol. 2021;74:1087–96. 33276032 10.1016/j.jhep.2020.11.038 PMC8062912

[3] Chappell LC Bell JL Smith A. Ursodeoxycholic acid versus placebo in women with intrahepatic cholestasis of pregnancy (PITCHES): a randomised controlled trial. Lancet. 2019;394:849–60. 31378395 10.1016/S0140-6736(19)31270-X PMC6739598

[4] Ovadia C Seed PT Sklavounos A. Association of adverse perinatal outcomes of intrahepatic cholestasis of pregnancy with biochemical markers: results of aggregate and individual patient data meta-analyses. Lancet. 2019;393:899–909. 30773280 10.1016/S0140-6736(18)31877-4 PMC6396441

[5] Heuman DM. Quantitative estimation of the hydrophilic-hydrophobic balance of mixed bile salt solutions. J Lipid Res. 1989;30:719–30. 2760545

[6] Tang B Tang L Li S. Gut microbiota alters host bile acid metabolism to contribute to intrahepatic cholestasis of pregnancy. Nat Commun. 2023;14:1305. 36894566 10.1038/s41467-023-36981-4 PMC9998625

[7] Fu HZ Ho YS Sui YM Li ZS. A bibliometric analysis of solid waste research during the period 1993–2008. Waste Manag. 2010;30:2410–7. 20620038 10.1016/j.wasman.2010.06.008

[8] Wu F Gao J Kang J. Knowledge mapping of exosomes in autoimmune diseases: a bibliometric analysis (2002–2021). Front Immunol. 2022;13:939433. 35935932 10.3389/fimmu.2022.939433 PMC9353180

[9] Wen R Zhang M Xu R. COVID-19 imaging, where do we go from here? Bibliometric analysis of medical imaging in COVID-19. Eur Radiol. 2023;33:3133–43. 36892649 10.1007/s00330-023-09498-z PMC9996554

[10] Wang J Maniruzzaman M. A global bibliometric and visualized analysis of bacteria-mediated cancer therapy. Drug Discov Today. 2022;27:103297. 35654388 10.1016/j.drudis.2022.05.023 PMC9530009

[11] Guleid FH Oyando R Kabia E Mumbi A Akech S Barasa E. A bibliometric analysis of COVID-19 research in Africa. BMJ Glob Health. 2021;6:e005690. 10.1136/bmjgh-2021-005690 PMC8111873 33972261

[12] Zeng D Wang J Xiao B Zhang H Ma X. A bibliometric visualization analysis on vaccine development of coronavirus disease 2019 (COVID-19). Vaccines (Basel). 2023;11:295. 36851173 10.3390/vaccines11020295 PMC9959778

[13] Wu F Li C Mao J Zhu J Wang Y Wen C. Knowledge mapping of immune thrombocytopenia: a bibliometric study. Front Immunol. 2023;14:1160048. 37207211 10.3389/fimmu.2023.1160048 PMC10189105

[14] Zyoud SH. Research landscape on COVID-19 and liver dysfunction: a bibliometric analysis. World J Gastroenterol. 2023;29:4356–67. 37545639 10.3748/wjg.v29.i27.4356 PMC10401660

[15] Kwok HT Van M Fan KS Chan J. Top 100 cited articles in male breast cancer: a bibliometric analysis. Breast Dis. 2022;41:15–20. 34219705 10.3233/BD-201024

[16] Wang W Wang H Yao T. The top 100 most cited articles on COVID-19 vaccine: a bibliometric analysis. Clin Exp Med. 2023;23:2287–99. 36939968 10.1007/s10238-023-01046-9 PMC10026222

[17] van Eck NJ Waltman L. Citation-based clustering of publications using CitNetExplorer and VOSviewer. Scientometrics. 2017;111:1053–70. 28490825 10.1007/s11192-017-2300-7 PMC5400793

[18] Synnestvedt MB Chen C Holmes JH. CiteSpace II: visualization and knowledge discovery in bibliographic databases. AMIA Annu Symp Proc. 2005;2005:724–8. 16779135 PMC1560567

[19] Zupic I Cater T. Bibliometric methods in management and organization. Organ Res Methods. 2015;18:429–72.

[20] Williamson C Geenes V. Intrahepatic cholestasis of pregnancy. Obstet Gynecol. 2014;124:120–33. 24901263 10.1097/AOG.0000000000000346

[21] Geenes V Chappell LC Seed PT Steer PJ Knight M Williamson C. Association of severe intrahepatic cholestasis of pregnancy with adverse pregnancy outcomes: a prospective population-based case-control study. Hepatology. 2014;59:1482–91. 23857305 10.1002/hep.26617 PMC4296226

[22] Beuers U Trauner M Jansen P Poupon R. New paradigms in the treatment of hepatic cholestasis: from UDCA to FXR, PXR and beyond. J Hepatol. 2015;62(1 Suppl):S25–37. 25920087 10.1016/j.jhep.2015.02.023

[23] Larson SP Kovilam O Agrawal DK. Immunological basis in the pathogenesis of intrahepatic cholestasis of pregnancy. Expert Rev Clin Immunol. 2016;12:39–48. 26469633 10.1586/1744666X.2016.1101344 PMC4893793

[24] Shen Y Zhou J Zhang S. Is it necessary to perform the pharmacological interventions for intrahepatic cholestasis of pregnancy? A Bayesian network meta-analysis. Clin Drug Investig. 2019;39:15–26. 10.1007/s40261-018-0717-2 30357607

[25] Li J Chen J Lee PMY Zhang J Li F Ren T. Familial clustering of intrahepatic cholestasis of pregnancy: a nationwide population-based study in Denmark. Hepatology. 2023;78:389–96. 36815353 10.1097/HEP.0000000000000328

[26] Zhu Y Xu L Beejadhursing R Li F. Maternal and neonatal outcomes of intrahepatic cholestasis of pregnancy after in vitro fertilization. BMC Pregnancy Childbirth. 2024;24:44. 38191339 10.1186/s12884-024-06248-x PMC10773009

[27] Tian YH Xu CY Zhang L Shi DK Cappelli F Yin SS. Maternal exposure to per- and polyfluoroalkyl substances: implications for intrahepatic cholestasis of pregnancy and adverse birth outcomes. Expo Health. 2024;15:1–15.

[28] Chen SY Ahlqvist VH Sjöqvist H. Maternal intrahepatic cholestasis of pregnancy and neurodevelopmental conditions in offspring: a population-based cohort study of 2 million Swedish children. PLoS Med. 2024;21:e1004331. 38227577 10.1371/journal.pmed.1004331 PMC10790993

[29] Zeng WJ Hou YY Gu W Chen Z. Proteomic biomarkers of intrahepatic cholestasis of pregnancy. Reprod Sci. 2024;31:1573–85. 38177949 10.1007/s43032-023-01437-z PMC11111573

[30] Li XY Liang XJ Gu XX. Ursodeoxycholic acid and 18β-glycyrrhetinic acid alleviate ethinylestradiol-induced cholestasis via downregulating RORγt and CXCR3 signaling pathway in iNKT cells. Toxicol In Vitro. 2024;96:105782. 38244730 10.1016/j.tiv.2024.105782

[31] Ye NZ Shi XR Gao JY. Exosomes from intrahepatic cholestasis of pregnancy induce cell apoptosis through the miRNA-6891-5p/YWHAE pathway. Dig Dis Sci. 2024;69:1253–62. 38361148 10.1007/s10620-023-08265-w

[32] Arthuis C Diguisto C Lorphelin H. Perinatal outcomes of intrahepatic cholestasis during pregnancy: an 8-year case-control study. PLoS One. 2020;15:e0228213. 32074108 10.1371/journal.pone.0228213 PMC7029845

[33] Keitel V Vogt C Häussinger D Kubitz R. Combined mutations of canalicular transporter proteins cause severe intrahepatic cholestasis of pregnancy. Gastroenterology. 2006;131:624–9. 16890614 10.1053/j.gastro.2006.05.003

[34] Wang S Feng R Wang SS. FOXA2 prevents hyperbilirubinaemia in acute liver failure by maintaining apical MRP2 expression. Gut. 2023;72:549–59. 35444014 10.1136/gutjnl-2022-326987

[35] Denson LA Bohan A Held MA Boyer JL. Organ-specific alterations in RAR alpha: RXR alpha abundance regulate rat Mrp2 (Abcc2) expression in obstructive cholestasis. Gastroenterology. 2002;123:599–607. 12145812 10.1053/gast.2002.34758

